# Governing Oneself Through HIV Diagnosis to Antiretrovirals

**DOI:** 10.1177/10497323231159804

**Published:** 2023-03-07

**Authors:** Nkululeko Nkomo

**Affiliations:** 1 Department of Psychology, School of Human and Community Development, 208666 University of the Witwatersrand, Johannesburg, South Africa

**Keywords:** HIV diagnosis, subjectivity, antiretrovirals, self-determination, adherence, responsibility

## Abstract

The article focuses on the experience of reinventing oneself post HIV diagnosis when living on antiretrovirals. Six women and men enlisted for antiretrovirals in South African public health facilities were interviewed, and a qualitative analysis was conducted drawing on Foucault’s theory of governmentality. For the participants, the prevailing governing rationality of taking personal responsibility for their health is synonymous with self-recovery and restoration of self-determination. From the hopelessness and despair of HIV diagnosis, for all six participants, committing to antiretrovirals enhances their capacity to take back control of their transformation from victim to survivor, and with it, a sense of personal integrity. Yet, an unwavering resolve to use ARVs is not always possible for some of them, is not preferable or is not always desirable, which perhaps signals that for certain people living with HIV, their life-long journey of self-governance with ARVs is likely to be characterized by constant contradictions.

## Introduction

In thinking about interventions for HIV/AIDS both in South Africa and elsewhere, it is striking how much they have evolved since the discovery of the epidemic in the early 1980s. Arguably, the development in the 1990s of more effective HIV treatments – especially the introduction of several antiretroviral medicines deployed either to decelerate the rate of HIV replication or prevent vertical transmission of HIV from mother to child – has significantly improved the life prospects of many people living with HIV (PLHIV) and altered the course of the epidemic around the world (see Heywood, 2009; Mykhalovskiy & Rosengarten, 2009). Though coordinated global efforts and lobbying to scale up access to antiretroviral therapy or ARVs in low- and middle-income countries prevented an estimated 4.2 million deaths between the years 2002 and 2012, HIV treatments were at first out of reach for most PLHIV in sub-Saharan Africa and many other regions of the developing world (WHO, UNICEF & UNAIDS, 2013).

In part, the reasons for lack of access included the high costs of the medicines, the limiting restrictions of international patent laws to make way for the production of more affordable generic antiretrovirals, and the absence of the political wherewithal or commitment to fostering a credible response to HIV/AIDS (Biehl, 2004; Geffen, 2010; Mbali, 2013). There were countries that endeavoured tenaciously to overcome these barriers. Soon after these medicines were introduced in 1996, for example, Brazil introduced an HIV treatment programme in the country’s public health system, a decision that managed to curtail considerably its rate of HIV infection and mortality (Iqbal, 2009). Brazil’s very rapid move in this direction was a very clear sign of the high level of political commitment to universal healthcare by the country’s political leadership, especially during the presidential tenure of Fernando Henrique Cardoso, from 1995 to 2003 (Galvão, 2005). Such a stance initially involved the country importing generic anti-HIV drugs from India and Thailand, even at the risk of violating international patent agreements, but later also undertaking to develop its own capacity to produce the drugs (see Biehl, 2007; Cataldo, 2008). Thus, ‘this policy of biotechnology for the people’, as Biehl (2004, 105) aptly described it, was embraced as an exemplary approach for other developing countries to emulate and emerged at the time as ‘an important component of international medical activism’. Within sub-Saharan Africa, Uganda and Botswana stand out for their early initiatives to introduce ARVs in their public healthcare systems, with the former enacting a plan to prevent the transmission of HIV from mother to child or PMTCT in the late 1990s, and the latter, in 2001, introducing a programme to offer a general ARV programme in the public health system (D’ Adesky, 2004).

In contrast, the battle for ARVs was hard won in South Africa. In 2001, after civil society groups took legal action against the ministry of health, the Pretoria High Court made a ruling compelling it to extend and provide PMTCT in the public health sector (see Kistner, 2003; Heywood, 2003, 2009; Nattrass, 2007, 2008; Mbali, 2013). The following year, a legal and civil campaign by AIDS lobby groups, most notably the Treatment Action Campaign (TAC), managed to put pressure on the government, under the stewardship of former president Thabo Mbeki, and the then Minister of Health, the late Dr Manto Tshabalala Msimang, to promulgate an operational plan for the care, management and treatment of HIV/AIDS.

As a result of the delays in South Africa in rolling out PMTCT and in the provision of ARVs to people who are eligible but too poor to buy HIV treatments, it is estimated as many as 330,000 people died between the years 2000 and 2005, with 35,000 babies born HIV-positive (Chigwedere et al., 2008). South Africa today continues to make progress in rolling out ARVs to PLHIV. Out of 7.5 million South Africans living with HIV, over 4 million of them are on ARVs, making the country’s public HIV treatment programme the largest in the world (Browne et al., 2019). The upscaling of ARVs in South Africa has also seen a rise in research to identify the barriers and facilitators to ARV adherence, the main objective behind it being to mitigate the behavioural risks to anti-HIV drug resistance (see Bartlett et al., 2009; Kagee et al., 2011). Beyond this instrumental objective, often overlooked in the research on ARV treatment adherence in the sub-Saharan African context is that the very nature and measure of value for PLHIV has been transformed by the availability of ARVs (Pollack Petchesky, 2003), reconfiguring their ethics, anxieties and aspirations (Venkatapuram, 2011).

This article critically examines the everyday subjective experience of living on antiretrovirals after HIV diagnosis. Such an undertaking arises from the premise that the contours of subjectivity are set or demarcated by social discourses (Butler, 1997), including prevailing health and social discourses. The interest of the article is the prescribed voluntary relations of rule linked to the ways PLHIV are enjoined to constitute and relate to themselves through their lived day-to-day experience with ARVs. This focus encapsulates both the aspects of governability and self-governance (see Cruikshank, 1999; Foucault, 2000; Rose & Miller, 1992).

## Using Governmentality as a Conceptual Tool

In this article, I take the approach that subjectivity has an instrumental role in enabling power relations, in so far as the potential to be spurred into action by others, including driving oneself, is underpinned by the idea of our own freedom to act independently (Foucault, 2000). The framework of governmentality conceptualizes all the facets that make for subjective conception and enactment as constituted through the nexus between power and knowledge. Therefore, subjectivity is the interiorization, or in Dean’s (1996) framing, the ‘enfolding of authority’ comprised in the manifold discursive positions made available through the various institutional and programmatic activities or initiatives based on the underlying rationale of securing human and population well-being (Rose, 2008).

The perspective adopted in this article is that subjectivity is produced, transformed and lived according to a set of rationalities and directives, including health advice and public health policy, laying out and expressing codes of conduct or action. Subjectivity is an instrument and effect of mentalities of government broadly defined, or ‘governmentality’, encompassed in all kinds of reflections, analysis, initiatives and programmes linked to advancing and protecting all things material and non-material pertaining to human existence (Lemke, 2015). According to Swyngedouw (2005), mentalities of government have their most effect and visibility in the production of selves that are governable, including their impact in spurring or driving self-governance.

Such a conceptual, and ultimately analytical, perspective, according to Foucault (1993, 203), focuses primarily on accounting for the structure of self-determination or agency, or how ‘technologies of domination of individuals over one another have recourse to processes by which the individual acts upon himself’, with the acting individual ‘integrated into structures of coercion or domination’. The intersections of a particular set of discursive practices that derive their particularity from the entirety of the practices in which they are articulated establish a structure of human subject self-determination (Adlam et al., 1977). The creation of ‘desiring selves, sexed selves, working selves, thinking selves, and intending selves capable of functioning as subjects’ is what Rose (1998, 172) refers to as the ‘production of subjectivity’. This shaping impact becomes apparent when multiple discursive processes are combined.

More important for the discussion in this paper is that these practices encapsulate, if not constitute, ideas on appropriate and effective means of mitigating or managing social, political and health problems, with the aim being to improve the overall standard of living and safeguarding ‘life itself’ (Vallgårda, 2015). In this sense, the notion of subjectivity as both related to the working of government and its outcome, at least from a Foucauldian perspective, presupposes a relationship with oneself premised on being directed by others as well as the self-turning implied in governing oneself (see Burchell, 1993; Cruikshank, 1993, 1994).

The framework or epistemology of governmentality largely offers a way to understand or think about the modes of rationality, valuation or ethicalization within which subjectivities are imagined and constructed (Dean, 1996). It has been criticized for overlooking the way selves or subjectivities are actually lived out across space and time (see Binkley, 2009a, 2009b; Murray-Li, 2014). More often than not, in most governmentality studies, subjective life appears as wholly enwrapped by and an effect of discourse. For Brady (2014, 13), such ‘deterministic’ and ‘linear’ approaches overlook the role of ‘multiplicity and context’ in anchoring any form of constitutive discourse in subjectivity. The power of a discourse to effect subjective constitution or transformation is contingent on the context or circumstances of appropriation. Subjective life may move in directions unexpected or surpassing the constitutive discourses by which it is produced (see Butler, 1997).

In this article, I analyze the neoliberal governmentality-related idea that rational choices or actions made by an individual rather than in light of circumstances have an impact on health outcomes (see Ayo, 2012; Marmot, 2015). This idea is mirrored in discourses on public health and policy relating to ARVs in South Africa, which are appropriated and translated into modes of subjective constitution and behaviours pertaining to using ARVs. As Bond (2005) has previously shown, the adoption of neoliberal public policy in post-apartheid South Africa has affected a variety of policy sectors, including the evolution of health policy. A Foucauldian governmentality conceptual framework was applied in this study in order to highlight the shape of the participants’ power relationship with themselves and how it is affected by public health and policy discourses around ARV therapy (see Cruikshank, 1993). The study thus concentrated on the various means through which policy and public health discourses on ARVs are appropriated and transformed into modes of subjective constitution and performance in everyday life as well as through the ordinary experiences and behaviours of HIV patients (see Gordon, 1991; Graham, 2005).

A deeper understanding of the subtle and complex ways in which people with HIV experience their connection to treatment and what shapes it from the perspective of the discourses underpinning this connection as well as the life circumstances that unravel it will fill an important but underappreciated knowledge gap in the literature. These narratives are the main emphasis of the research goal and the study’s inquiry.

## Method

### Selecting the Data for Analysis

Six interview narratives of women and men accessing antiretroviral therapy from the public health system were analyzed. First-person accounts open the window to exploring how policy and public health discourses are reproduced daily in quotidian personal strategies for living on ARVs, indeed their anchoring in subjectivity. Semi-structured interviews were conducted. This interview method or technique was chosen because it allows the participants to bring their understanding or reflection upon the experience being investigated (Babbie & Mouton, 2001). Ethics clearance for the study was granted by the Human Research Ethics Committee (Non-Medical) of the University of the Witwatersrand in Johannesburg.

The participants were recruited through the help of a key informant who was himself HIV-positive. Only participants over the age of 18 were recruited for the study. They also had to have known that they are living with HIV for more than 3 years. With their own background with HIV, the hope was that with these recruitment criteria, willing participants will have the knowledge, if not experience, of the South African HIV/AIDS landscape that preceded and followed the roll-out of ARVs. The small sample size made it possible to engage more deeply with the participants’ personal accounts of HIV infection, the consequences and experiences that followed, up to their journey with ARV therapy. All the interviews were conducted by the author and took place at the facilities of a community-based AIDS advocacy organization in Pimville, one of the 26 cluster townships of Soweto in Johannesburg, South Africa. All of the participants had children, whose ages ranged from toddlers to teenagers. At the time of the interview, the six participants were Soweto residents. They were either home-based caregivers or members of the organization’s support group when they had a relationship with the AIDS advocacy group. The youngest person to be interviewed was in her late twenties and the oldest was in her late 40s. At the time of the interviews, between 2012 and 2013, all the participants were receiving ARVs from their local public clinic or hospital. With the exception of the one participant who had started a few months prior to the interview, most had been enrolled for ARVs for at least a period of 3 years or longer.

The study was explained to the participants before each interview, and they had the chance to ask any questions they might have if they wanted further explanation. They were also informed about the consent procedure for the study. All of the participants provided written approval for both their participation and the interview’s recording. Out of respect for their right to privacy, the identities of the participants are not utilized in this study; instead, they will be made anonymous based on the order of the interviews, from interview 1 to interview 6.

### Data Analysis

Open coding was employed initially, followed by connecting the codes and combining inductive and deductive analysis. Coding for the interview transcripts was done line-by-line and subsequently grouped into significant themes and sub-themes (Yin, 2010). The existing literature (see Decoteau, 2014; Pienaar, 2016; Robins, 2004, 2009) was especially helpful for identifying the emergent and broad themes of the struggle for ARVs and in the post-struggle or in the so-called ‘post-AIDS’ (see Dowsett & McInnes, 1996) landscape with respect to the significance of ARVs in altering personal attributions around the experience of living with HIV. The analysis was also open to adopting any new themes and sub-themes that were coded from the transcripts (Yin, 2010). This assisted in ensuring that coding is not manipulated but is rather adapted to the realities or experiences relayed in the interview transcripts. This analytic approach was best suited to gather both a broad and the depth of understanding necessary from the interview transcripts analyzed. The conceptual framework of governmentality was integrated into the analysis process through the coding procedure and the themes and sub-themes that were found by concentrating on the participants’ willing participation in ARV therapy and the beneficial ways they carry out their own self-governance (see Graham, 2005). The discussion of the findings is presented below.

## Refusing to Be a Victim: Taking Back Control

The six participants spoke of the transformative value of ARVs in terms much like being born again. For this group of participants, the memory of the struggle for ARVs was still fresh in their minds; they understood the significance of having access to ARVs, not only politically but also personally. For them, enrolling for ARVs was a commitment they were making to themselves and significant others to engage in the ongoing work of refiguring their HIV subjectivity and taking responsibility for their treatment outcomes.

### Resignification of HIV

This sub-theme investigates how the experiences of people who have HIV might illuminate how neoliberal governmentality shapes behaviour or conduct through both emotions and ‘practical rationalities’ (D’ Aoust, 2014). These feelings show how the ARV programme has helped the participants develop an emotional connection to their responsibilities. In other words, we also need to recognize that governmentality processes, as D’ Aoust (2014, 267) has argued, ‘are concomitant with the enclosure and valorisation of certain subjective/emotional dispositions’ in order to comprehend why or how the participants have come to value their responsibilization. As we will see later, the mediational function of ARVs relies on the resignification of HIV, which transforms the initial sensation of hopelessness into one of empowerment. The attachment to accountability and self-responsibility highlighted by the participants in the next sub-theme reflects this emotional transformation that ARVs have caused in the participants’ subjective metamorphosis.

To demonstrate this shifting influence, the stories of *#Interviewees 1* and *2* will be recounted. *#Interviewee 1* is an HIV-positive gay man who was in his mid-30s when he was interviewed. When he tested HIV-positive, his perception was that ‘…there wasn’t a difference between HIV and AIDS’. This perception had a significant impact on his outlook:

…you immediately just told yourself that you’ve got AIDS...you just told yourself that 1, 2, 3, you’re dead ... I didn’t love myself. I hated myself. I started doing bad things…I gave in to alcohol. I never used to drink, at all. I never smoked...I gave in to alcohol…So…eish (emotional pause).

The altered perspective of himself is one of fatalism, self-abjection, lack of self-regard. His perspective was not uncommon among PLHIV before ARVs were made available. A number of studies have documented the despair and feelings of hopelessness in the experience of HIV before ARVs (see Pienaar, 2016; Robins, 2004, 2009; Rohleder & Gibson, 2006). *#Interviewee 1* underwent what Nel and Judge (2008) refer to as ‘corrective rape’, which he experienced as a gay man and which resulted in his seroconversion to HIV in the late 1990s. His perspective was indeed profoundly shaped by this traumatic experience. In his account later in the interview, he reflected on the role of ARVs in empowering him, allowing him to ‘somewhat’ loosen the grip of the rape terror that was still so deeply inscribed in his body and consciousness, and so intense, that it had once limited any vision for his life.

For *#Interviewee 2*, who was in her early 40s at the time of the interview, the shock of testing HIV-positive immediately gave way to a feeling of insecurity around public spaces out of the fear of being seen as HIV-positive. After ‘…I got my results’ during a routine test for HIV when she was pregnant in the late 1990s, she felt not just that when ‘I looked at myself I did not understand…I wanted full proof that, yes, I was HIV-positive’. She described feeling ‘as if I had been written off – as if everyone in the taxi knew my status’ as she travelled back home on the day she received her test results. In the way that *#Interviewee 1* and *#Interviewee 2* described their lives before ARVs, HIV was damaging for their self-identity. Shame was another way they also felt about themselves.

An HIV diagnosis does constitute an event that generates an immense sense of loss and disruption that gives rise to feelings of vulnerability and hopelessness (see Parker & Aggleton, 2003; Rohleder & Gibson, 2006). Accessing ARVs provided *#Interviewee 1* and *#Interviewee 2* the very grounds to re-signify HIV, restore hope and work at self-recovery. As stated by Rose-Spratt (2021, p. 11), healthism requires ‘a performance of health-oriented lifestyles and behaviors that present the person engaging with them as a “good citizen” by revealing their compliance with, and support of, neoliberal ways of thinking about individualism and personal responsibility’. ARVs enabled *#Interviewees 1* and *2* to begin this journey on their own. The similarity in reaction of the two participants to their HIV diagnosis must be contextualized within the period of the global HIV/AIDS epidemic when HIV carried the association of a death sentence, rather than a manageable chronic illness that it is today.

### Accountability and Responsibility

Participants in the study emphasized the value of independence and self-control in terms of their way of life, behaviour and health, shedding light on the pervasive influence of terms used by neoliberal governmentality to describe the practice of placing the onus of responsibility back on the individual as several earlier and more recent studies have shown in relation to individual health practices in countries of the Global North (see Ayo, 2012; Rose-Spratt, 2021; Sweet, 2018).

Given their frequent interactions with healthcare professionals, the participants’ interactions presented value for taking ARVs – personal accountability or acting in one’s own best health interest – is one that they are urged to live by. In fact, for some people on ARVs, a loss of self-respect and self-care quickly changed into an opening of hope for living. *#Interviewee 6* praised the ability to manage oneself that ARVs provide, saying: ‘I do not see how I will fail if I take ARVs for the rest of my life. After all, I want to live as long as possible for my sake, the sake of my kids, and the sake of my family’. *#Interviewee 6* was in her late 40s at the time the interviews were conducted, making her the oldest participant to be interviewed. She had discovered she has HIV while pregnant. ARVs have allowed her to regain control over her life for not only her own sake but also for that of her children and family. For her, turning away from helplessness after HIV diagnosis meant identifying unequivocally with the responsibility of using ARVs:

They said at the clinic that if I take these pills today, for instance, I will know the following day how I respond to them. Should I feel uncomfortable about them the next day, I should return them and get them changed.

For *#Interviewee 6*, taking control of HIV is accomplished through the small-scale struggles of the everyday realities of living with HIV, including her willingness to follow the urging by her healthcare providers to have her ARV regimen substituted for another one should the one she is taking not work for her. The implication is that there is really no choice for her, except to follow the health advice if she wants to live a long life and be around for her children and family while living with HIV.

*#Interviewee 6* views personal responsibility as involving the ability to counter any fixation with victimhood, engaging and adopting an outlook towards oneself and the world of self-determination: ‘Many people just sit with their discomforts and think the pills are somewhat being effective, when they are not. No, it is not that they are not effective, but that there is something wrong with them. So, people sit with them, making them dizzy and so on without going to the clinic. And you go the second time for treatment, but you still don’t raise the issue and the side effects become worse. Had you highlighted these effects, they would have done something about the pills. The people at the clinic know how these pills work’. The enactment of such responsible behaviour in the perspective of *#Interviewee 6* should be a matter of routine everyday living, built into a way of understanding, assessing and acting on oneself when enrolled onto ARVs. An unwillingness or inability to assert your personal agency, by not raising any difficulties you have with taking ARVs, for her is disempowering. It is also a way to preserve her sense of self where she is of many receiving the service.

Such a view is based on the unstated presumption that enrolling onto ARVs does not automatically guarantee the prospect of prolonging one’s life, in and of itself, unless it is augmented by accountability and due diligence. The transition to ARVs as a strategy for reviving the hopes of PLHIV is made more significant and justifiable by this necessity. One could even assert, in line with Ayo (2012), that this participant conceptualization demonstrates how neoliberal governmental health discourses are infused into South African policy guidelines for ARVs (see National Department of Health, 2004, 2020). It acts as a reminder to carefully follow the directions for starting ARV therapy and a warning against deviating from the suggested schedule.

Let us go back to *#Interviewee 1*. Adherence to ARVs is non-negotiable for him, if not unconscionable: ‘if you’re supposed to drink them at eight, four hours shouldn’t pass if you forgot to drink them at that time…to this day, I still do that’. Not only does his life depend on ARVs; they have empowered him, too. At the time of the interview, he was an HIV/AIDS activist in his community. This kind of self-discipline or stoicism by *#Interviewee 1* is not only a way to elevate ‘my CD4 count, which is currently very high’, of boosting ‘my immune system’, but ‘when your immune system is high, that’s when you feel as if you’re succeeding in life…’ Declaring it with conviction, for *#Interviewee 1*, ‘ARVs have given people life, especially those who are like me with positive minds…’ There is also the physical and psychological recovery for him:

They also decrease your clinic visits as you are not sick as often as you were before. You become healthy and go back to square one, before you fell sick. That’s what made me realise that ARVs are the best.

Adopting the strategy and attitude toward ARVs advised by health practitioners helps one avoid clinic visits turning into a regular occurrence, according to *#Interviewee 1*. This helps one bravely and actively fight against regressing to a condition of helplessness. From the perspective of someone like *#Interviewee 1*, taking ARV adherence seriously is about self-reclamation after the trauma of HIV diagnosis. Therefore, it is in terms of the prospect of assuming control over health outcomes that for a participant like *#Interviewee 1* and *#Interviewee 6* that ARVs have raised personal accountability as a criterion of judgement and evaluation in both the personal and collective struggle against HIV/AIDS. Being accountable for the participants is, in fact, a personal guiding concept to live by for navigating daily life on ARVs and is described as therapeutic or health citizenship in the governmentality literature (see Mfecane, 2011; Robins, 2009; Rose, 2007). It was also crucial to the TAC’s operation of the *Treatment Literacy Campaign* in Lusikisiki in the Eastern Cape Province and Khayelitsha in the Western Cape Province, which took place prior to the launch of the nation state’s ARV programme in 2004 (Geffen, 2009, 2010). All six participants endorsed it and said they lived by it; however, for some of them, it did not always comfortably align with other daily duties or expectations.

## Beyond Responsibility Only: The Perils of Self-Determination

Every day, policy and scientific discourses certainly construct enrolment onto ARVs as involving cost/benefit trade-offs, with implications for constraining economic, social or aesthetic choices (see Mfecane, 2011; Persson, 2004, 2005). In such ways of understanding the processes of negotiating a life-long commitment to ARVs, there will inevitably be complex compromises entailed in enlisting for ARVs and sticking to prescribed regimens. Health and policy discourses place more emphasis on physical survival, often underplaying the real fears and concerns behind delays to enrolment and inadequate adherence to ARVs (Becker et al., 2020; Heestermans et al., 2016; Kagee et al., 2011). The next three participants talked about how the imperative of absolute dedication to ARVs sometimes encompasses trade-offs and feelings of ambivalence on the part of those to whom it is directed.

### Working Through Conflicting Selves

*#Interviewee 5*, who was in his late 20s when interviewed and diagnosed with HIV in 2000, when he had a recurring skin problem, was very clear that ARVs have made a world of difference for his general well-being and particularly his skin condition. While the fear of stigma or discrimination was less of a challenge for him having disclosed his HIV status to everyone he knows, he still talked about the psychical ambivalence that comes with depending on ARVs for managing HIV. The challenge is that at the clinic, ‘they do mention the fact that you should not drink alcohol whilst you’re on ARVs, but as you know, you’re still young, you want to hang out with the guys’. Being on ARVs for him feels like being ‘caught’ between two worlds: enjoying his youth and being conscious of the limits to his enjoyment because he is on ARVs.

At this point in the interview, there was a sadness in his eyes. ARVs make him uncertain about how to freely be himself with his friends, even with their knowledge and acceptance of his HIV status:

It’s as if you’re living on them, it’s hard…Sometimes you’re hanging out with someone in your shack and its 21:00 and you have to drink with them, now you have to hide yourself. I have to make excuses to be able to drink with them. It’s tough...it’s tough. It’s difficult living with the fact that at a certain time there are these pills you have to take.

Between these conflicting expectations of himself, he ends up placing more value on his health: ‘but this is your life, there is nothing you can do, you have to take the pills’. For *#Interviewee 5*, there is then the pressure of living with the limits to his personal autonomy and having to reevaluate his ideas of who he is or should become when on ARVs.

*#Interviewee 4*, who was in her late 20s when she was interviewed, valiantly postponed starting on ARVs until she had absolutely no choice. She discovered that she is HIV-positive when she became very ‘seriously ill’ in 2002. In her words, diagnostic test results showed that ‘my CD4 count had dropped to 164. I told them at the clinic I would not take them. I did not have a child then. Better that I die than take ARVs, I had thought, you see’. However, she was forced to start on ARVs when she became pregnant ‘for the sake of living for my child’, but even then, she ‘still remained stubborn’ and ‘procrastinated’. She felt that she ‘was not ready’ until she became very ‘sickly and no longer enjoyed life’. When asked for a reason for her resistance to starting on ARVs, this was her response:

People were saying these pills make you mad; that they change your shape; people would say so and so’s shape has changed because of these pills.

Persson and Newman (2006) found in their study in Australia that HIV-positive gay men on ARVs associated the iatrogenic effects of ARVs with a non-idealized, non-normalized or undesirable bodily state that could invite public attention. ARVs similarly evoked a return to a form of unwelcome visibility for *#Interviewee 4*. She was also anxious that ARVs could set off undesirable body shape changes for her, an issue that we see in her response:

Someone told me that her shape has changed owing to these pills…when I looked at her body, I also noticed that it was out of shape. So, I preferred dying to having my shape change. At that time, I was still interested in possessing a good shape. But then I changed my attitude and accepted the treatment, only because I did not have a choice. I took the treatment, and experienced none of the shape related side effects.

*#Interviewee 4’s* account highlights most forcefully the importance of another fear about the effects of ARVs – disintegration of a valued body image of oneself. Clinging to her body shape image was perhaps also *#Interviewee 4’s* way of maintaining a continuity between who she is post HIV diagnosis and who she was before HIV diagnosis. Both the stories of *#Interviewee 4* and *#Interviewee 5* encapsulate the continuing journey towards subjective transformation and renewal that never ends at the point of starting on ARVs. Between these seemingly irreconcilable selves of the two participants, one or the other at different times might have to give in depending on where or what someone is going through at that point in their life journey with HIV.

### Difficult Choices

Like all the participants, in his account, *#Interviewee 3* was very clear that he is devoted to taking ARVs responsibly, although sometimes the devotion is breakable. A father of three children who was in his mid-40s at the time of the interview, he was diagnosed with HIV in 2000 after a persistent cough that would not go away. For him, a diagnosis of HIV was less disheartening than it had been for some of the participants that had been interviewed, maybe owing to the fact that he was immediately enrolled onto ARVs after first being on treatment for tuberculosis and his kidneys were subsequently compromised. *#Interviewee 3* clearly recognized the huge importance of ARVs for his well-being: ‘No, there are things I can forget, not them. Sometimes though, 8:30 am passes without my taking them and then I would drink them at 10. True, I would have forgotten to take them at 8:30 am, but then I would take them eventually’. When he was asked about what ARVs mean to him and for his sense of living with HIV, without equivocation, he gave the response that ‘they saved my life a lot’.

However, as he later noted, ‘Once I stopped taking them for a week, just to see what would happen. After a week I began to cough a lot, and then lost weight’. When the interviewer probed him for an explanation for this decision, he admitted that ‘the thing is...it is like this…when your CD4 count is less than 200, (and you know there are no jobs now) you could go and register for a disability grant, you see. And then you get it. Then you can support your child and you yourself can eat; you see. Now, if the CD4 count is more than 200, you get no grant. But you have to take these pills, and these pills you take for life. Now you have no job…So I thought I should stop taking these pills, to reduce my CD4 count. That’s why I had stopped taking these pills’. From a governmentality standpoint, the ARV programme urges self-discipline and personal responsibility, or what Foucault (1993) refers to as ‘technologies of the self’, but ignores the limited control that many people have over their capacity to do so (Marmot, 2015). In *#Interviewee 3’s* case, this may lead to an over-individualization of the problem rather than addressing the structural injustices that make some people more likely to not always take ARVs. Accordingly, altering one’s behaviour could make following rules seem straightforward, but for many people, their behaviour is established due to a lack of choice, which is ultimately caused by events beyond their control. #Interviewee 3 was willing to make the trade-off between not taking ARVs and incurring the danger of jeopardizing his health and physical well-being because he is unemployed and has no other source of income.

In his account, there is also recognition by *#Interviewee 3* that ‘Looking at it carefully, it hurts to reduce your CD4 count for the sake of money’. It is like he is betraying what he believes is correct for his health, his responsibility to himself and the others who depend on him living longer by adhering to the life-saving medication. Yet, it is equally ‘painful to take these pills without food…because of unemployment’. If it is possible to conquer the temptation to stop taking the ARVs, that is what *#Interviewee 3* has attempted unsuccessfully. He was struggling to find work. In his perception, his age prevented him from finding employment: ‘they ask you how old you are and when you are above 30, they don’t take you’. He continued to say that ‘when you go register for a disability grant, they say you don’t qualify. You then go and see the doctors, they say “stay on that treatment, permanently”’. These challenges undermined his ability for adherence to ARVs. In the end, he had this to say: ‘but how do you take these pills when you have had no supper’. When the choices for him were between taking ARVs or receiving a disability grant, even at the risk of reducing his CD4 or compromising his health, economic or financial survival has won out before. More concerning is that there is absolutely no guarantee that while he remains unemployed, or unless there is a change in the social grants’ policies for PLHIV, he will not make a similar trade-off again in the future.

## Discussion

In order to raise awareness of the concerns of PLHIV and assert their equal right to healthcare by receiving the life-saving medication from public healthcare facilities, the fight for ARVs in South Africa turned to a discourse that emphasizes victimization characterized by hopelessness (see Butler, 2005; Cameron, 2005; Geffen, 2009, 2010; Heywood, 2003, 2009; Mbali, 2013). The claim was based on the logic that accessing ARVs will afford PLHIV their agency, and this will help them in divesting from a victim-based identity (see Kistner, 2003; Robins, 2004, 2009). In various ways, the participants in this study embody this claim in their journey from HIV victims to survivors through ARVs. In the interviews, they reported that they feel unencumbered by HIV and free to reclaim who they were and who they want to be with ARVs making it possible for them. This illustrates how neoliberalism, as governmentality, encourages individual behaviours by the way people perceive and treat themselves (see Graham, 2005). In this regard, if policy is a fundamental tool of government, it depends on the self-management or self-entrepreneurialism of the people it affects for it to have an effect and be made operational (see Gordon, 1991; Cruikshank, 1994, 1999).

Furthermore, the study’s findings demonstrate that the participants are aware of and willing to accept the responsibilities that come with living independently on ARVs (see Cruikshank, 1999; Foucault, 2000; Rose, 1998). To use Dean’s (1996) language, the study showed that the discourse of responsibilization is ‘enfolded’ by the participants as a sought and beloved ideal that promises relief from the effects of HIV and a practical way to regain control of their life. This viewpoint is in line with neoliberal governmentality (see Ayo, 2012; Swyngedouw, 2005) in that stopping ARV therapy could be viewed as a conscious decision rather than the result of contextual constraints that prevent some participants from acting in their own best health interest by continuing to fully take the life-saving medication. Indeed, the interviewees’ commitment to ARVs and responsible use of them is not immaculate, as some of their testimonials from the interviews revealed. For this reason, it is critical to emphasize the significance of ‘multiplicity and context’, as Brady (2014) would put it, in determining the capacity for self-management, particularly with regard to health-related self-care, in governmentality research. The goal of this study was to shed light on the concomitant subjectivity and complexity that ARV therapy patients face in relation to their treatment.

## Conclusion

The subjective daily experience of being on antiretrovirals after being diagnosed with HIV was studied in this article. Through analysis, the article showed how current health and social discourses have an impact on participants’ participation with and self-management of ARV therapy. This area of focus encompasses both self-governance and governability.

The right to healthcare and dignity should be guaranteed to everyone, whether or not they are HIV-positive (see Biehl, 2004, 2007, D’ Adesky, 2004; Galvão, 2005; Iqbal, 2009; Venkatapuram, 2011). This is the guiding premise around which the global fight for ARVs was based. The fact that the ARV programme is dependent on people’s abilities and decisions is much more significant.

The likelihood of non-adherence increases when that occurs because a disabling sense of helplessness and impotence may manifest. Self-determination to follow ARV guidelines on a practical level can be paradoxical, exposing flaws and necessitating a realignment of behaviours. Interventions in health and social policy for PLHIV must consider the variety of aspects of lived experience as well as the significance of uncertainty in daily lives.
